# 635. 'Urine' the Know: A Simple Approach for Detecting Candida auris Sooner, and the Emergence of Clade III Candida auris in NY

**DOI:** 10.1093/ofid/ofaf695.199

**Published:** 2026-01-11

**Authors:** Maya Polashenski, Francois Lebreton, Jason Bennett, Moein Mozafari, Jennifer West, Jason Stam, Robert Pence, Yoon Kwak, Patrick McGann, Emil P Lesho

**Affiliations:** Rochester Regional Health, Rochester, New York; Walter Reed Army Institute of Research, Silver Spring, Maryland; Walter Reed Army Institute of Research, Silver Spring, Maryland; Rochester Regional Health, Rochester, New York; Rochester Regional Health, Rochester, New York; Walter Reed Army Institute of Research, Silver Spring, Maryland; Rochester Regional Health, Rochester, New York; Walter Reed Army Institute of Research, Silver Spring, Maryland; Walter Reed Army Institute of Research, Silver Spring, Maryland; Rochester Regional Health, Rochester, New York

## Abstract

**Background:**

*Candida auris* (CA) poses an urgent public health threat due to its multidrug resistance and associated mortality. Compared to other clades, Clade III CA (CA-III) may display increased environmental persistence and transmissibility. To date, we found no reports of Clade III in New York State. We sought to describe the genomic and clinical epidemiology of CA-III after a dual hospital outbreak in upstate NY.

**Table 1:**
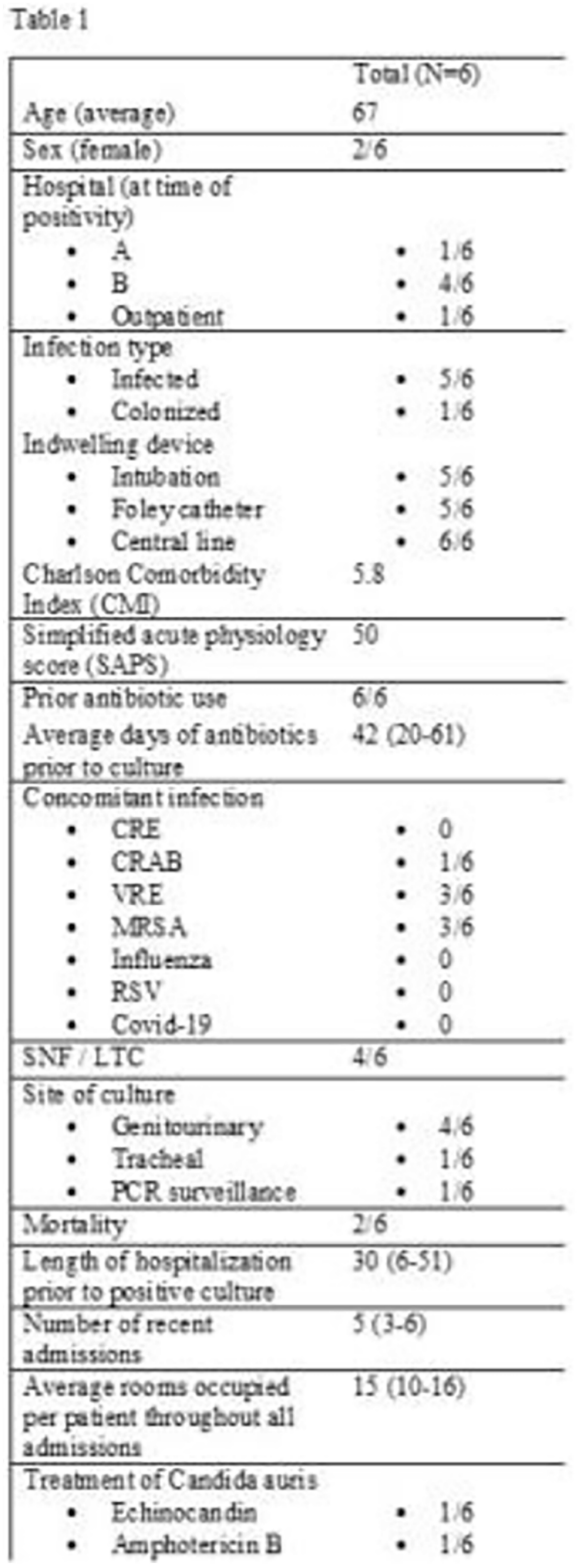
Candida auris baseline characteristics

**Figure 1:**
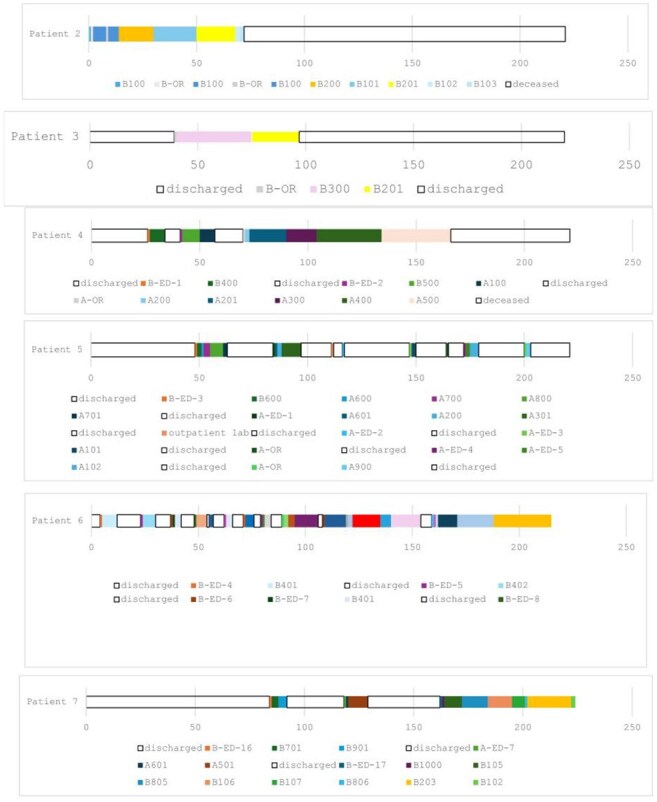
Hospital and room location of the Candida auris patients (A=hospital A; B=hospital B)

**Methods:**

Contact tracing and testing, environmental sampling, cleaning observations with intensified cleaning, and whole genome sequencing were performed. To minimize the risk of missing CA in mixed urine cultures, processing of routine urine cultures was modified such that all inpatient urine cultures yielding mixed results underwent an additional 24hr incubation at 35-37 °C, and any yeast isolated was identified to the species level.

**Table 2:**
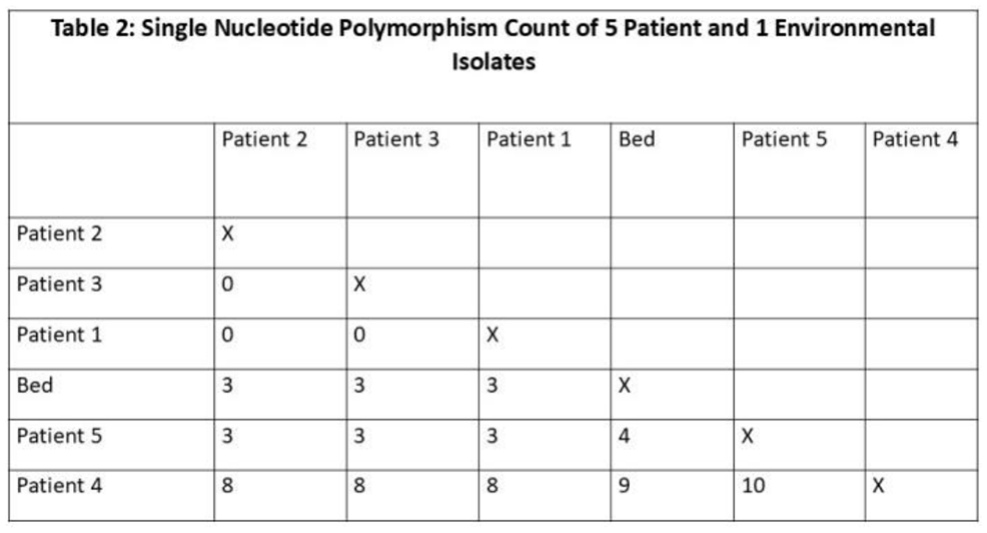
Single nucleotide polymorphism count of 5 patient and 1 environmental isolates

**Results:**

Case (n=6) characteristics appear in Table 1. 67% of cases were discovered via the modified urine protocol. On average, each patient had five prior admissions and 15 different rooms per stay (Table 1, Figure 1). All had invasive devices and considerable antibiotic exposure (Table 1). The outbreak began in the operating room, and later a common ward at hospital A became the epicenter of future cases. All isolates were highly related, differing by < /= 10 SNPs, including the only positive environmental culture from a bed (Table 2). Direct cleaning observations revealed that surface disinfectants were not given sufficient contact time on several occasions.

**Conclusion:**

Our findings demonstrate the usefulness of a laboratory modification that is readily adaptable by most hospitals and does not require additional resources such as swabs, a PCR assay, or referral laboratory support. Moreover, unlike PCR screening, the urine protocol allows for susceptibility testing and WGS of isolates. This report re-demonstrates the role of antibiotic exposure in the emergence of CA. It also underscores the challenges of contact tracing given the frequent room transfers and re-admission rates typical among patients prone to CA acquisition. Last, our report highlights the role of shared equipment in transmission, and the criticality of appropriate cleaning and disinfectant use.

**Disclosures:**

All Authors: No reported disclosures

